# A mental health-informed, online health promotion programme targeting physical activity and healthy eating for adults aged 60+ years: study protocol for the MovingTogether randomised controlled trial

**DOI:** 10.1186/s13063-022-06978-3

**Published:** 2022-12-27

**Authors:** Chiara Mastrogiovanni, Simon Rosenbaum, Kim Delbaere, Anne Tiedemann, Scott Teasdale, Annaliese McGavin, Nancy Briggs, Grace McKeon

**Affiliations:** 1 Discipline of Psychiatry and Mental Health, University of New South Wales, Sydney, NSW 2052 Australia; 2 School of Health Sciences, University of New South Wales, Sydney, NSW 2052 Australia; 3 Falls, Balance and Injury Research Centre, Neuroscience Research Australia, University of New South Wales, Sydney, NSW 2031 Australia; 4 School of Population Health, University of New South Wales, Sydney, NSW 2052 Australia; 5 Institute for Musculoskeletal Health, The University of Sydney and Sydney Local Health District, Camperdown, NSW 2050 Australia; 6 Mindgardens Neuroscience Network, Sydney, NSW 2052 Australia; 7 School of Psychology, University of New South Wales, Sydney, NSW 2052 Australia; 8 Stats Central, Mark Wainwright Analytical Centre, University of New South Wales, Sydney, NSW 2052 Australia

**Keywords:** Mental health, COVID-19, Isolation, Physical activity, Falls, Older adults

## Abstract

**Background:**

The COVID-19 pandemic and associated social distancing regulations have led to an increased risk of social isolation and physical inactivity, particularly among older adults. The benefits of physical activity for reducing fall risk and improving mood and mental functioning have been well documented. The aim of this trial is to investigate the effect of the *MovingTogether* programme on psychological distress (primary outcome) and physical activity, social capital, cognition, concern about falling, loneliness, physical functioning, quality of life and physical activity enjoyment (secondary outcomes).

**Methods:**

A randomised controlled trial with a waitlist control will be conducted, recruiting 80 adults aged 60+ years with access to Facebook and a computer or tablet and not currently meeting the aerobic physical activity guidelines. Randomisation will be completed using REDCap. The intervention group (*n* = 40) will join a private Facebook group where allied health facilitators will provide targeted healthy lifestyle education throughout the 10-week programme with weekly telehealth group calls. Intervention participants will also be provided access to tailored strength and aerobic exercise guidance and an evidence-based eHealth balance exercise programme. Psychological distress and secondary outcomes will be assessed at baseline, 11 weeks (post-intervention) and 16 weeks (1-month follow-up). Linear mixed models will be applied for each outcome measure as per an intention-to-treat approach to determine the between-group differences. Secondary analyses are planned in people with greater adherence and those with higher psychological distress.

**Discussion:**

COVID-19 has highlighted the need for scalable, effective and novel methods to improve and protect the health of older adults. The integration of an evidence-based fall prevention programme with a mental health-informed online health promotion programme may help to improve mental and physical health outcomes among older adults.

**Trial registration:**

Australian New Zealand Clinical Trials Registry (ANZCTR) ACTRN12621001322820p. Registered on 29 September 2021

**Supplementary Information:**

The online version contains supplementary material available at 10.1186/s13063-022-06978-3.

## Administrative information

Note: The numbers in curly brackets in this protocol refer to the SPIRIT checklist item numbers. The order of the items has been modified to group similar items (see http://www.equator-network.org/reporting-guidelines/spirit-2013-statement-defining-standard-protocol-items-for-clinical-trials/).

**Table:** 

Title {1}	A mental health-informed, online health promotion programme targeting physical activity and healthy eating for adults aged 60+ years: study protocol for the MovingTogether randomised controlled trial
Trial registration {2a and 2b}.	ACTRN12621001322820p (Australian New Zealand Clinical Trials Registry, ANZCTR) on 29 September 2021.
Protocol version {3}	Protocol Version 1.0 1 July 2021.
Funding {4}	This research received no specific grant from any funding agency in the public, commercial or not-for-profit sectors.
Author details {5a}	^1^ Discipline of Psychiatry and Mental Health, University of New South Wales, Sydney, NSW 2052, Australia ^2^ School of Health Sciences, University of New South Wales, Sydney, NSW 2052, Australia ^3^ Falls, Balance and Injury Research Centre, Neuroscience Research Australia, University of New South Wales, Sydney, NSW 2031, Australia ^4^ School of Population Health, University of New South Wales, Sydney, NSW 2052, Australia ^5^ Institute for Musculoskeletal Health, The University of Sydney and Sydney Local Health District, Camperdown, NSW 2050, Australia ^6^ Mindgardens Neuroscience Network, Sydney, NSW 2052, Australia ^7^ School of Psychology, University of New South Wales, Sydney, NSW 2052, Australia ^8^ Stats Central, Mark Wainwright Analytical Centre, University of New South Wales, Sydney, NSW 2052, Australia
Name and contact information for the trial sponsor {5b}	Dr Ted Rohr (UNSW), ted.rohr@unsw.edu.au.
Role of sponsor {5c}	On behalf of UNSW, the trial sponsor takes responsibility for the initiation and management of the clinical trial.

## Introduction

### Background and rationale {6a}

Older adults, classified as people aged 60+ years [1], are at an increased risk of experiencing health issues such as mental health problems and falls [2]. Psychological distress increases one’s risk of falling due to the demanding use of the working memory that is also needed for activities requiring complex attention and coordination [3]. Similarly, experiencing depressive symptoms is a risk factor for falling in older adults, independent of other factors like antidepressant use and reduced executive and physical functioning [4]. Given the bi-directional relationship between falls and mental health, interventions aiming to reduce fall risk may simultaneously improve mental health outcomes, and vice versa [5].

Falls are the leading cause of morbidity and mortality among older adults and can lead to ongoing physical disability, fear of falling, social isolation and loneliness [6, 7]. There is strong evidence that specific exercise that includes a challenge to balance is effective in reducing the rate of falls among older people living in the community [8]. Achieving physical activity guidelines of 150 min of moderate to vigorous activity [9] has been recommended to manage mental health conditions and is protective against incident depression in adults aged > 60 years [10]. Additionally, physical activity is protective against osteoporosis, all-cause and cardiovascular mortality, certain cancers, disability, functional limitation, cognitive decline and dementia [11].

Due to the COVID-19 pandemic, there has been a decline in social connection [2] as well as physical activity among older adults [12]. Older adults are more likely to be self-isolating than the general population, often for extended durations due to comorbidities and government recommendations [13], therefore increasing the risk of psychosocial issues for older adults. Loneliness is a risk factor for depressive symptoms in those aged over 50 [14], and decreased social connection is another modifiable risk factor for older adults being more vulnerable to loneliness, poor mental health and decreased quality of life [2]. Subsequently, older adults face an increased risk of depression, anxiety and stress-related disorders in response to the pandemic [15].

Isolation in older people is not only limited to pandemics and can occur when people are limited by disease comorbidities or even the weather. It is therefore important to find better mechanisms to address reduced physical activity levels and social connectedness for older adults during extended periods of isolation. *StandingTall* is an evidence-based, eHealth fall prevention programme that includes a progressive balance and strength training programme plus health education that has demonstrated efficacy for the rate of falls over 2 years [16, 17]. Adherence to the recommended dose of 2 h per week was high throughout the study period, with limited attrition, but there was no effect on psychological well-being [17]. A qualitative study further highlighted that while participants valued being able to do the exercises in their own time at home, many participants also highlighted the lack of social connections as a limitation of the programme [18]. We hypothesise that the addition of specific strategies that target mental health, through a social media platform, will address this limitation and improve its effectiveness for mental health.

We recently conducted a pilot study that evaluated a health promotion programme delivered via a private Facebook group to eleven socially isolated older adults during the COVID-19 pandemic in 2020 [19]. The programme was adapted from a Facebook-delivered, mental health-informed physical activity programme that was effective at reducing psychological distress in emergency service workers and their support partners [20, 21]. Similarly to emergency service workers and those in their support system, older adults are at risk of poor mental health, so the programme was then modified to have targeted content for older adults. The pilot study illustrated feasibility, high acceptability and preliminary effectiveness for secondary outcomes including psychological distress and quality of life [19].

### Objectives {7}

This randomised controlled trial (RCT) will enhance the design of the programme used in the pilot study by adding *StandingTall*, creating the *MovingTogether* programme. It is hypothesised that *MovingTogether* will improve both mental health outcomes, primarily psychological distress and physical health outcomes.

### Trial design {8}

A two-arm, superiority RCT will be conducted with participants randomly assigned (1:1) to either the intervention group (*n* = 40) or the wait list control group (*n* = 40). The design of this protocol was guided by the Standard Protocol Items: Recommendations for Interventional Trials (SPIRIT) checklist [22] and the Template for Intervention Description and Replication (TIDieR) checklist (Table 1) [29]. Reporting of the results from this trial will be guided by the Consolidated Standards of Reporting Trials (CONSORT) checklist [30].

**Table 1:** Template for Intervention Description and Replication (TIDier) checklist for the *MovingTogether* trial protocol

Item number	Item	Description
1	Brief name	An online health promotion, physical activity and social support, *MovingTogether* programme for older adults.
2	Why	Due to the COVID-19 pandemic, there has been a decline in social connection and physical activity in older adults, a population that is already at an increased risk of experiencing health issues such as mental health problems and falls. Given the bi-directional relationship between falls and mental health, interventions aiming to reduce fall risk may simultaneously improve mental health outcomes, and vice-versa. Physical activity is one strategy that can improve mental health and decrease fall risk, and using technology for service delivery is an emerging area of research for mental healthcare.
3	What materials	• Weekly educational written and video posts delivered in the *MovingTogether* private Facebook group (see Table 2). • *StandingTall* eHealth fall prevention programme. • Participants may use a folded towel (substituted for a foam cushion) for *StandingTall* exercises. • Tailored aerobic and strength exercise programmes including pictures and written descriptions. • Participants who choose to join weekly group telehealth calls will use Zoom, a freely available live call application. • Facebook is a freely available social media platform that participants and facilitators will use to communicate.
4	What procedures	When participants gain access to the 10-week programme, they will join a private Facebook group. The group will be used for social support and communication and to deliver healthy lifestyle education to participants. The facilitators will tailor aerobic and strength exercise programmes for each individual and instruct participants on the use of *StandingTall* balance exercises. Optional weekly group video calls will also be available.
5	Who provided	The facilitators are members of the research team who are allied health professionals (Accredited Exercise Physiologists and an Accredited Practising Dietitian). Allied health facilitators will have experience working with people with mental health conditions and/or older adults. At least one member of the facilitating team will be required to have their mental health first aid accreditation.
6	How	There are both group and individual activities in the programme that will be completed online.
7	Where	The trial will be completed online, using social media (Facebook), video calls (Zoom) and an eHealth fall prevention programme (*StandingTall*).
8	When and how much	Over the 10-week programme, the duration and frequency that individuals complete their *StandingTall* exercises and aerobic and strength training will vary depending on individual circumstances and goals. However, the facilitators will guide most participants to reach 2 h per week of *StandingTall* exercises, at least 150 min of moderate to vigorous intensity aerobic exercise and two sessions of muscle strengthening per week.
9	Tailoring	Accredited Exercise Physiologists will design tailored exercise programmes for all individuals depending on their health conditions, functional ability, interests and goals. Similarly, the *StandingTall* website regularly re-evaluates each individual’s balance performance and adjusts the intensity and level of challenge accordingly.
10	Modifications	Not applicable as the trial intervention has not been delivered yet.
11	Planned intervention adherence assessment	Facilitators will be recording how many participants view each educational Facebook post. Adherence to *StandingTall* training and individual aerobic and strength programmes will also be monitored. Participants will be contacted privately by study investigators if they choose not to attend calls for at least two consecutive weeks and/or if they do not reach their recommended exercise dose for two consecutive weeks.
12	Actual intervention delivery	Not applicable as the trial intervention has not been delivered yet.

## Methods: participants, interventions and outcomes

### Study setting {9}

All participants will be living in Australia, although the trial will be completed entirely online.

### Eligibility criteria {10}

Individuals will be considered eligible if they meet the following inclusion criteria: (i) aged 60+ years; (ii) proficient in written and spoken English; (iii) living independently in the community, in Australia; (iv) able to mobilise indoors without the use of a walking aid; (v) have access to the Internet at home, Facebook and either computer, laptop or iPad/tablet access; and (vi) self-reported to be currently participating in less than 150 min of moderate to vigorous intensity physical activity per week through the use of the Physical Activity Vital Sign Questionnaire [31].

Individuals will be excluded from the study if (i) they have high levels of psychological distress (score of > 30 on the K10) unless deemed to have support from a mental health professional or (ii) they have a high risk of suicidal behaviour (score of > 21 on the SIDAS) unless deemed eligible to participate by a clinical psychologist through a suicide risk assessment. Other exclusion criteria are (i) absolute contraindications to exercise according to the American College of Sports Medicine (ACSM) or relative contraindications without medical clearance to exercise [32], (ii) currently participating in a fall prevention programme at least weekly, (iii) signs of cognitive impairment (score < 4) as assessed by the Ottawa 3DY or (iv) diagnosis of a progressive neurological condition.

### Who will take informed consent? {26a}

If participants confirm they would like to be involved after reading the participant information statement and consent form, they will be emailed a link to provide online consent and to complete screening questionnaires via Research Electronic Data Capture (REDCap). REDCap is a secure web platform that will be used by facilitators to collect data throughout the trial [33]. Alternatively, upon request, participants may be screened and complete future trial questionnaires via a phone call with the primary study investigator, initiated by verbal consent.

### Additional consent provisions for collection and use of participant data and biological specimens {26b}

Not applicable, no samples were collected.

### Interventions

#### Explanation for the choice of comparators {6b}

A waitlist control design allows all participants to eventually have access to the programme.

#### Intervention description {11a}

The *MovingTogether* intervention has three essential components, i.e. a Facebook-delivered healthy lifestyle education and support programme, an eHealth fall prevention programme (*StandingTall*) and an aerobic and strength exercise programme tailored to each participant.

Participants will be asked to join the *MovingTogether* private Facebook group. Facilitators will monitor the group at least once every weekday over the 10-week period. Targeted education and resources will be provided to participants through the online Facebook group, and facilitators will encourage discussion between all members of the group, regarding a variety of lifestyle topics. Topics will include behaviour change, overcoming barriers, reducing sedentary behaviour, increasing physical activity, increasing structured exercise, balance training and healthy eating (see Table 2). Education on a single topic will be the focus of a single week (2 weeks in the case of the nutrition topic), and new material will be released every week. These educational materials have been designed specifically for this study by the facilitators who are allied health professionals (Accredited Exercise Physiologists and an Accredited Practising Dietitian) and will be delivered as written resources or videos. Allied health facilitators will have experience working with people with mental health conditions and/or older adults. At least one member of the facilitating team will be required to have their Mental Health First Aid Accreditation. Participants will be encouraged to discuss shared experiences with participation in the programme and more general life experiences and to support each other. Additionally, participants can choose to join an optional weekly 20–30-min group video call [19], to further promote the social connection between participants and to follow up and create discussion about the provided educational materials. Facilitators will run calls twice weekly to allow multiple time options to join.

**Table 2:** *MovingTogether* weekly intervention content

Week	Topics	Content
1	Introductions, safety and *StandingTall*	○ Participants invited to introduce themselves ○ Participants directed to the *Safe Exercise at Home* safety checklist [23] ○ Group set up of *StandingTall* and individual baseline assessment led by AEP through a live video call
2	Starting *StandingTall*	○ Prompted to start using the *StandingTall* programme
3	Exercise education	○ Education on aerobic, resistance and balance training and how often to complete these based on the physical activity guidelines [9]
4	Behaviour change	○ Goal setting education and participants to create personal goals in a style that suits the individuals [24] ○ Forming and tracking habits ○ Invited to talk to AEP about progressing *StandingTall* use
5	Nutrition	○ Live video call with an Accredited Practising Dietitian including: - Fuelling the body with good nutrition [25, 26] - Creating a healthy food environment, for example, mindfully eating meals - Nutrition for mental health, brain health and fall prevention ○ Individual diet investigation through the University of Newcastle’s Healthy Eating Quiz [27, 28]
6	Nutrition	○ Live question and answer session with an Accredited Practising Dietitian
7	Barriers and follow-up on behaviour change	○ Facebook poll used to reflect the specific barriers to exercise and barriers to healthy eating faced by the individuals in the group ○ Suggested strategies to address barriers ○ Follow-up on goal setting ○ Invited to talk to AEP about progressing *StandingTall* use
8	Starting aerobic and resistance exercise	○ Participants encouraged to determine what level of exercise is suited to them based on the provided *Safe Exercise at Home* guide [23] ○ Exercise ideas shared for all different levels through documents and real-time videos ○ Individuals invited to privately speak to an AEP about a tailored aerobic and strength exercise programme
9	Reducing sedentary behaviour	○ The risks of sedentary behaviour and the importance of breaking up sitting time ○ Suggested ways to break up sitting time
10	Review	○ Individualised approach to maintaining aerobic, strength and *StandingTall* exercise ○ Recommendations to online and community programmes ○ How to access a private exercise physiologist ○ Review of goals ○ Celebration of progress

The *StandingTall* eHealth programme provides home-based exercise to improve balance and reduce fall risk [16]. Individuals will be provided with access to the programme and a manual on setting up and using the application. They will join a small group video call to receive assistance from a facilitator to set up the app and will be encouraged to contact the primary study investigator for assistance with any technological difficulties or queries. The app will provide participants with an individualised exercise programme that will suit their abilities, but remains challenging, based on a baseline balance assessment. This assessment will be completed in week 1 when the participants are guided to set up their *StandingTall* account. The programme will be progressed automatically based on the reported rate of perceived exertion through training. Exercises will train static and dynamic balance, stepping in different directions, functional strength and dual-task activities. Frequency, duration, intensity and dose of *StandingTall* training will be individualised to participants, but participants will be guided by facilitators to set goals around completing their exercise programmes with the aim of encouraging all to gradually complete a recommended dose of 2 h of balance training per week. Website analytics will track adherence data and time spent on the app data through individual logins.

Participants will also receive individualised support from allied health facilitators, including tailored aerobic and strength exercise programmes prescribed by Accredited Exercise Physiologists depending on participants’ health needs and activity interests. Exercise programmes will be designed to encourage participants to reach the physical activity guidelines of 150 min of moderate to vigorous activity, unless the participant indicates during goal setting that this volume is unrealistic for their abilities and/or circumstance [9].

At the conclusion of the 10-week programme, the Facebook groups and *StandingTall* programme will remain accessible without input from facilitators. The waitlist control group will also have access after their final assessment at 14 weeks from baseline. They will join a private Facebook group (separate from the intervention group) and complete the facilitated intervention without answering additional questionnaires. Based on feedback from the intervention group, the researchers will be using the waitlist control programme to trial new features of the programme such as additional topics suggested.

#### Criteria for discontinuing or modifying allocated interventions {11b}

Participants who experience any change to their health during the trial that precludes exercise will still be permitted to receive the non-exercise-related components of the intervention; however, their data will be excluded from the analysis, as exercise is a key component of the intervention.

#### Strategies to improve adherence to interventions {11c}

Participants will be contacted privately by study investigators if they choose not to attend calls for at least two consecutive weeks and/or if they do not reach their recommended exercise dose for two consecutive weeks. This is to ensure they receive support to reach their goals and to promote adherence to the exercise components of the programme.

#### Relevant concomitant care permitted or prohibited during the trial {11d}

Participation in another fall prevention programme for the duration of the trial will not be permitted.

#### Provisions for post-trial care {30}

If participants become upset or distressed as a result of participation in the research project, the research team will be able to arrange for counselling or other appropriate support. Any counselling or support will be provided by qualified staff who are not members of the research team. This counselling will be provided free of charge. Alternatively, a number of free contactable support services are included on the participant information sheet.

### Outcomes {12}

The primary outcome is psychological distress, and secondary outcomes include physical activity levels, social capital, cognition, concern about falling, loneliness, quality of life, physical functioning and exercise enjoyment. Participants will complete all questionnaires at baseline, week 11 (post-intervention) and at the 1-month follow-up point (see Table 3) so that change from baseline to post-intervention and change from baseline to the follow-up point can be evaluated. It will take approximately 45 min for participants to complete questionnaires at each time point. A demographics questionnaire will also be completed by participants at baseline only. This will ask participants about their age, the highest level of education completed, marital status, living situation, employment status, known medical conditions, their mobility around the house and outdoors, fall history, self-reported concern about falling and Facebook usage.

**Table 3:** Timeline for data collection

	Pre-programme		Programme	Post-programme	
	Enrolment (< − 1 week)	Baseline (− 1 week)	Weeks 1–10	End of week 10	Follow-up (+ 4 weeks)
**Eligibility screen**					
Demographics questionnaire	X				
PA vital sign	X				
Ottawa 4DY	X				
SIDAS	X				
Informed consent		X			
**Assessments**					
Pre-programme tool		X			
K10	X	X		X	X
IPEQ		X		X	X
Cognitive Brain Games Battery		X		X	X
Social Capital Questionnaire		X		X	X
EQ-5D		X		X	X
UCLA Loneliness Scale		X		X	X
Functional component of the Late Life Function and Disability Instrument		X		X	X
Icon-FES		X		X	X
PACES		X		X	X
**Adherence**					
Average weekly *StandingTall* training duration (recorded by the app and monitored following data transfer to the server)				X	
Total *StandingTall* training duration (recorded by the app and monitored following data transfer to the server)				X	
Facebook post views (recorded by Facebook)				X	
Average weekly training frequency and duration of individualised aerobic and strength programmes (participant survey)				X	

*PA*, physical activity; *Ottawa 4DY*, Ottawa ‘day, date, world, year’; *SIDAS*, Suicidal Ideation Attributes Scale; *K10*, Kessler 10; *IPEQ*, Incidental and Planned Exercise Questionnaire; *European EQ-5D*, Quality of Life five dimensions; *Icon-FES*, Iconographic Falls Efficacy Scale; *PACES*, Physical Activity Enjoyment Scale

### Participant timeline {13}

**Fig. 1:**
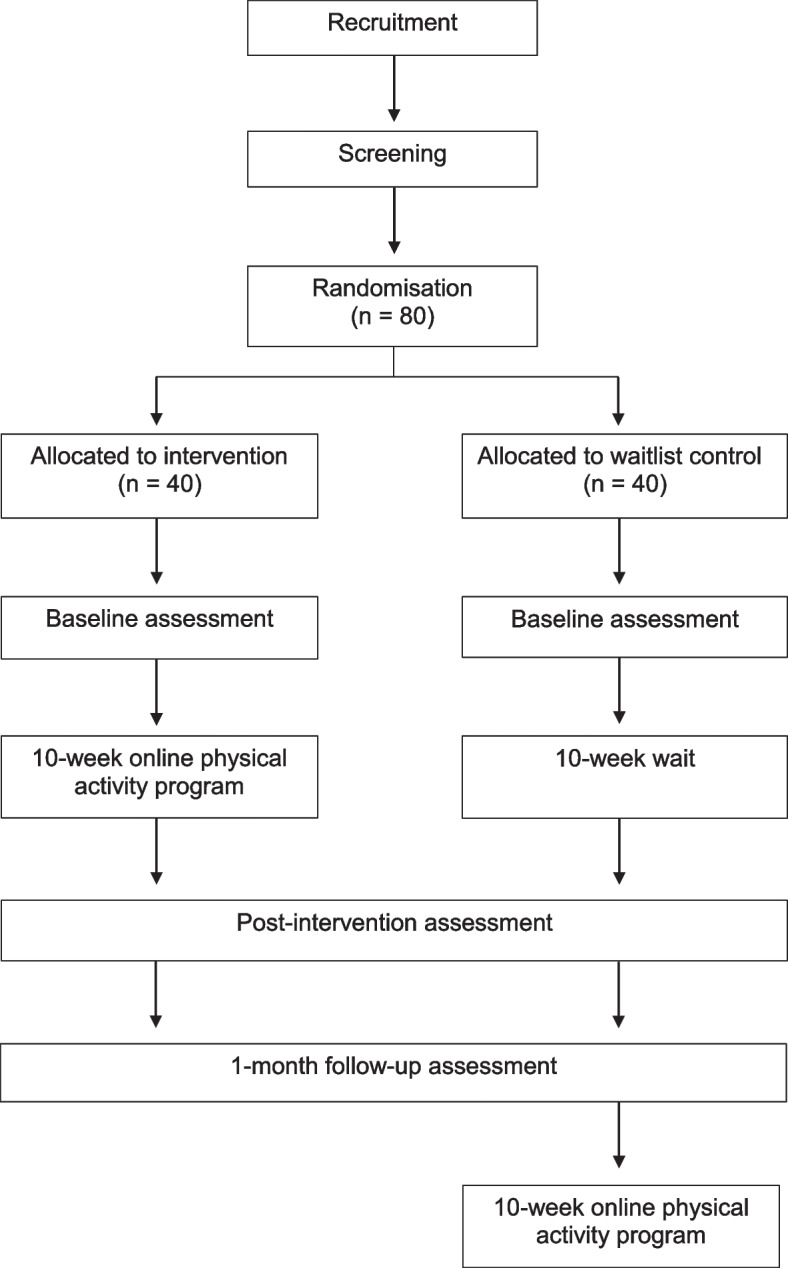
Chart shows the participant flow through the study and intervention process

Figure 1 illustrates the participant flow through the trial.

### Sample size {14}

#### Power calculation

A power analysis was conducted using GLIMMPSE [34] based on pilot data [19] to estimate the variance for this larger study [35]. To get an estimate of the variance of psychological distress (K10), the sum for the three time point (pre, post and follow-up) K10 measures were calculated. Then, a mixed model with common error variance for the time points and a compound symmetric covariance matrix was run.

A sample size of *n* = 68 is required, assuming SD = 6.41, correlation between repeated values of 0.6 and 80% power to detect a moderate effect. Allowing for a dropout rate of 15%, the total sample required is *N* = 80. This sample size is comprised of the following samples from each of the participant groups:

1. Participant group 1 (intervention): 40
2. Participant group 2 (waitlist control): 40

#### Recruitment {15}

Participants will be recruited through targeted online and print advertisements. The recruitment advertisement will also be posted on the social media accounts of study investigators, Facebook advertisements and news outlets targeting the 60+ year age group. Interested individuals will respond through a website called JotForm. They will follow a link to a private webpage and by entering their contact details into a form, consent to being contacted by the study investigators to provide a participant information statement and consent form.

#### Assignment of interventions: allocation

##### Sequence generation {16a}

Randomisation will use randomly permuted block sizes of 2, 4 and 6 within an online REDCap database. Participants will be allocated (1:1) to either the intervention or waitlist control group after they have been screened for eligibility, provided consent and completed all baseline measures. Participants in the same household will be considered one unit for this process.

##### Concealment mechanism {16b}

Computer-generated randomisation will ensure concealed group allocation.

##### Implementation {16c}

An investigator not involved with recruitment and the screening process will set up a randomisation schedule. Additionally, assessors conducting the statistical analyses will be blinded to group allocation.

### Assignment of interventions: blinding

#### Who will be blinded {17a}

The investigators setting up a randomisation schedule on REDCap and conducting statistical analysis will be blinded to participant identities. Trial participants will not be blinded to the intervention.

#### Procedure for unblinding if needed {17b}

Not applicable, participants will be aware of the group that they have been allocated to.

### Data collection and management

#### Plans for assessment and collection of outcomes {18a}

Data will be collected through REDCap surveys and via videoconferencing for one-on-one interviews. Email notifications from REDCap will invite participants to access questions at the appropriate time points. Data will be collected through REDCap [33] (or via phone call if the participant prefers) and a computer software called Inquisit 6.

#### Primary outcome

##### Psychological distress

The K10 provides a score indicating one’s self-reported psychological distress [36]. Five possible responses are provided as answers, with corresponding numerical values. Total scores range between 10 (no distress) and 50 (severe distress). The K10 has excellent psychometric properties, including high internal consistency (Cronbach’s alpha 0.93 [36] and discriminant validity [37]).

#### Secondary outcomes

##### Physical activity

The Incidental and Planned Exercise Questionnaire (IPEQ) has been designed specifically for older people and comprises 10 items that prompt users to self-report the frequency and duration of planned and incidental physical activities. This questionnaire has excellent psychometric properties [38]. The results provide minutes across different categories of incidental and planned activities, e.g. sport, walking.

##### Social capital

The Brief Social Resources Questionnaire (Annaliese McGavin, Registered Psychologist & UNSW School of Psychology PhD Candidate: Brief Social Resources Questionnaire 2021, unpublished) summarises the participants’ self-reported responses to questions about the context of their social capital, in particular, the objective and subjective aspects of their social resources. Three social network questions—adapted from the Lubben Social Network Scale-6 [39]—reveal a more objective and quantitative measure of their interactions, while the three questions surrounding social support—adapted from Duke Social Support Index-10 [40]—focus on the qualitative, perceived impact of those interactions. Adaptations of existing questionnaires were made to capture both objective and subjective aspects of social capital with brevity. Responses to each of the six items are standardised.

##### Cognition

Multiple aspects will be assessed through a battery of ‘brain games’ (Annaliese McGavin, Registered Psychologist & UNSW School of Psychology PhD Candidate: Brain Games 2021, unpublished) delivered through the Inquisit 6 web application [41]. Four tasks will evaluate decision-making related to risk-taking and uncertainty (Iowa Gambling Task) [42], working memory (n-Back task) [43], cognitive flexibility (Trail-making test) [44] and emotional intelligence (EI) (Trait Emotional Intelligence Questionnaire (TEIQue)) [45]. Each task has demonstrated adequate reliability and validity for measuring each aspect of cognition.

##### Concern about falling

The Iconographical Falls Self-Efficacy Scale (Icon-FES) is used to evaluate concerns about falling. The Icon-FES is a 30-item questionnaire that uses pictures as well as words to ask about concerns about falling doing 30 common daily activities. This scale has high internal consistency and excellent test-retest reliability [46].

##### Loneliness

Loneliness and social isolation is evaluated through the 20 questions of the University of California, Los Angeles (UCLA) Loneliness Scale [47]. Questions are answered on a 4-point Likert scale ranging from ‘never’ to ‘often’. A total score over 25 represents a high level of loneliness and 30 or higher indicates a very high level of loneliness. This scale has been deemed reliable and valid when assessing loneliness in a population over 65 years [47].

##### Quality of life

The European Quality of Life Five Dimensions (EQ-5D-5L) gauges details about an individual’s mobility, self-care, usual activities like work, study, housework and leisure activities, pain and/or discomfort and anxiety and/or depression. Each dimension is scored based on one item. The tool also includes a visual analogue scale (VAS) that users rate their health status ranging from 0 (worst possible health status) to 100 (best possible health status) [48]. The EQ-5D-5L has excellent psychometric properties for a broad range of populations and conditions [49].

##### Physical functioning

The functional component of the Late Life Function and Disability Instrument (LLFDI) is used [50]. Thirty-two items are investigated on a 5-point Likert scale where individual’s answer can range from ‘never’ to ‘often’. Lower scores reflect less trouble performing physical, functional tasks. Evidence suggests that the use of the LLFDI for community-dwelling older adults can assess functional limitations is valid with the high test-retest functional component [51].

##### Enjoyment

The Physical Activity Enjoyment Scale (PACES) comprises 18 questions that are intended to be answered on a 7-point bipolar rating scale [52]. The use of this tool has been validated in older adults [53].

#### Monitoring

Participant adherence to balance exercise training will be recorded. Average weekly training duration and total training duration will be recorded through automatic data transfer to a server [17]. The intensity of challenge is monitored using a self-report modified rate of perceived exertion scale to allow exercises to be adjusted with performance changes [17].

Other data to be collected includes the number of participants who viewed each educational Facebook post and the adherence to individualised aerobic and strength programmes. This will be assessed using a survey at the post-intervention assessment point asking participants the average frequency and duration of individualised programme completion per week.

#### Interviews

Participants will also be invited to participate in a 20–30-min one-on-one semi-structured interview via Zoom. The aim will be to gain practical feedback about how the programme can be improved. Feedback will then be applied when facilitators run the programme for the waitlist control group. Participants will be asked about their experience participating in the programme and what they perceived to be the programme’s strengths and weaknesses. Of those indicating interest in this optional interview, those with both the lowest and highest adherence to the programme will be interviewed first until data saturation is achieved [54]. This is to ensure a representative sample of participants is included. Interview transcripts will be used for thematic, qualitative analysis [55].

Bias will be reduced with the use of a control group and random group allocation. This will allow a causal effect to be established for the accurate evaluation of primary and secondary outcome measures.

#### Plans to promote participant retention and complete follow-up {18b}

At data collection points, participants will receive an email invitation from REDCap to complete questionnaires. In the absence of a response, participants will receive a follow-up reminder.

#### Data management {19}

Data will be stored on the secure UNSW OneDrive and will be kept for 15 years after publication. A full data management plan can be found on UNSW’s ResToolKit, Research Data Management Plan ID H0241934.

#### Confidentiality {27}

Information about participants will be stored in a re-identifiable format where any identifiers such as name, address and date of birth will be replaced with a unique code. Only the listed researchers will have access to participant data.

#### Plans for collection, laboratory evaluation and storage of biological specimens for genetic or molecular analysis in this trial/future use {33}

Not applicable, no samples will be collected.

## Statistical methods

### Statistical methods for primary and secondary outcomes {20a}

Data analysis using IBM SPSS 25 will evaluate whether there are any significant changes between baseline and post-intervention on psychological distress and secondary outcomes, as well as between baseline and results at the follow-up point. Linear mixed models will be applied for each outcome measure, excluding physical activity, conducted using the MIXED procedure (considering data at all time points). A generalised linear mixed model with appropriate distribution will be used to analyse physical activity, measured by the IPEQ. Time will be considered a categorical variable for each outcome. Effect sizes (Cohen’s *d* and the 95% confidence intervals) will be calculated to determine the size of the within-group change between before and after treatment and between before treatment and 1-month post-intervention. Time, group and the interaction between these will be used as predictors. Marginal means drawn from the mixed models will be used.

Analyses will use all available data as per an intention-to-treat approach. The between-group difference at post-intervention and follow-up from baseline [mean difference (MD) and 95% confidence interval] will be calculated for all outcome measures using a baseline-adjusted mixed model with group as the independent variable, post-programme (week 10) and also follow-up (week 14) score on the outcome measures as the dependent variables, baseline score on the outcome measure as a covariate, and statistical significance set at *P* < 0.05. The results and effects will be reported as unadjusted and adjusted for sex and age. Time, group and the interaction between these will be used as predictors.

### Interim analyses {21b}

Not applicable, no interim analyses are planned.

### Methods for additional analyses (e.g. subgroup analyses) {20b}

Subgroup analyses will be conducted on psychological distress for the following groups:

- Those who demonstrate mild or higher levels of psychological distress at baseline, indicated by a K10 score of 20 or above
- Participants who complied with the intervention, including those who reached a *StandingTall* training duration of at least 2 h per week
- Those who experienced at least one fall in the last year versus those who had not

Effect sizes (Cohen’s *d* and the 95% confidence intervals) will be calculated to determine the size of the within-group change between before and after treatment and between before treatment and 1-month post-intervention.

### Methods in analysis to handle protocol non-adherence and any statistical methods to handle missing data {20c}

A sub-group analysis is planned to include participants who complied with the intervention, including those who reached a *StandingTall* training duration of at least 2 h per week. We will report the number of participants with missing scores for each outcome.

### Plans to give access to the full protocol, participant-level data and statistical code {31c}

Participant data will not be made publicly available as the re-identifiable format allows participants to easily be identified.

### Oversight and monitoring

#### Composition of the coordinating centre and trial steering committee {5d}

The trial will be coordinated primarily by a research student investigator, who will be supervised by a post-doctoral researcher. Both are Accredited Exercise Physiologists based at UNSW. They will meet weekly prior to and during the trial to plan and discuss the progress of the trial.

#### Composition of the data monitoring committee, its role and reporting structure {21a}

The data monitoring committee will consist of three researchers who will be able to advise the researchers handling data on their responsibilities and procedures concerning data analysis and storage. Researchers on the data monitoring committee and researchers handling data will be independent from the trial sponsor and will have no competing interests.

#### Adverse event reporting and harms {22}

Participants will be strongly encouraged to inform the research team of any physical or mental harms caused by the programme, and the research team will report the adverse events to the UNSW Human Ethics Office within 72 h of becoming aware of the event.

#### Frequency and plans for auditing trial conduct {23}

Auditing of the trial will be conducted annually. The research team will fill in an annual monitoring form indicating trial status, adverse events, complaints and protocol deviations. This will be submitted to UNSW HREC.

#### Plans for communicating important protocol amendments to relevant parties (e.g. trial participants, ethical committees) {25}

Protocol modifications will first be submitted to the UNSW Ethics Committee for approval before implementation. If approved, trial participants will be informed of changes through an email from the investigators.

#### Dissemination plans {31a}

The findings of this trial will be disseminated through peer-reviewed publication(s) and academic conference presentations. These strategies will target academics and mental health and exercise professionals. Further social media will be used to reach this audience as well as the general public.

## Discussion

The *MovingTogether* programme combines a mental health-informed online health promotion programme delivered via social media [19] with an eHealth exercise-based fall prevention [17] and general physical activity programme to improve mental health by increasing physical activity. Physical activity programmes have demonstrated improvements in many indicators of mental health such as quality of life, psychological distress, depression and anxiety [56]. Research has shown that to increase adherence to exercise among older adults, programmes should focus on group cohesion and social support and should provide individualised health benefits and tailored advice [57, 58]. Lifestyle changes, including a reduction in physical activity [12], due to social isolation (e.g. during the COVID-19 pandemic), have highlighted the need for scalable, effective and innovative methods of achieving improvements in both the mental and physical health of older adults. Using technology for service delivery is an emerging area of research for mental healthcare [59], and it has been established that eHealth can be effective at promoting physical health in older adults [60].

### Limitations

It is likely that most of our participant sample will be those who are considered technologically literate and willing to try something new. While it is important that participants can use Facebook and the *StandingTall* programme, it will be difficult to evaluate the uptake, adherence and effect that this programme could have on a wider population, including those who do not currently engage in social media use. We have partially addressed this by releasing recruitment materials through print media, alongside the online sources as the main method of recruitment. Additionally, the result of subgroup analyses including the effect of the programme on those with high psychological distress and those who have greater adherence to the programme will have limited power.

## Conclusion

This RCT will investigate the efficacy of *MovingTogether*, a combined Facebook-delivered healthy lifestyle education and support programme, an eHealth fall prevention programme and tailored exercise. The findings will be used to inform the delivery of a potentially scalable online programme for adults aged 60+ to improve mental and physical health.

## Trial status

Protocol version 1.0 (1 July 2021). Recruitment started on 7 February 2022 and was completed on 10 June 2022. Data collection was completed on 3 October 2022. The authors note that the protocol was submitted to *Trials* on 29 April 2022 prior to the enrolment of the last participant on 10 June 2022. However, the trial was registered on the Australian New Zealand Clinical Trials Registry (ANZCTR) (ACTRN12621001322820p) on 29 September 2021, before the start of recruitment.

## Supplementary Information

**Additional file 1:.** UNSW Insurance.

**Additional file 2:.** Ethical Approval.

**Additional file 3:.** Participant Information Sheet.

## Abbreviations

AEP: Accredited Exercise Physiologist
AQoL-6D: Assessment of Quality of Life
EI: Emotional intelligence
EPs: Exercise physiologists
ESSA APSS: Exercise Sports Science Australia Adult Pre-exercise Screening System
K10: Kessler 10
PA: Physical activity
PTSD: Posttraumatic stress disorder
RCT: Randomised controlled trial
RPE: Rate of perceived exertion
SIDAS: Suicidal Ideation Attributes Scale
SIMPAQ: Simple Physical Activity Questionnaire
