# Supplementary material for: A mental health-informed, online health promotion programme targeting physical activity and healthy eating for adults aged 60+ years: study protocol for the MovingTogether randomised controlled trial
Source: Trials. 2022 Dec 27;23:1052. doi: 10.1186/s13063-022-06978-3 (PMC9793388; doi:10.1186/s13063-022-06978-3)
Supplement: Supplementary file 1 — Additional file 1:. UNSW Insurance. [file 13063_2022_6978_MOESM1_ESM.pdf]

# Certificate of Currency

**Date of Issue:** 30 October 2020

To whom it may concern,

**Contact:** Shannon Dunn
Client Relationship Manager

**t:** +612 9253 7063
**e:** shannon.dunn@aon.com

**We hereby certify that the under mentioned insurance policy is current as at the date of this certificate, please refer to the important notices below.**

| | |
|---|---|
| **Policy Type** | Clinical Trials Insurance |
| **Insured** | University of New South Wales<br><br>Additional Insureds:<br>Kirby Institute;<br>UNSW Counselling Service and Compass Programs (UNSW Psychology Clinic);<br>UNSW Health Service (University of New South Wales Medical Centre); UNSW (Medicine) Lifestyle Clinic;<br>University of New South Wales Optometry Clinic<br>UNSW Sport and Recreation (UNSW Lifestyle Centre);<br>UNSW Psychology Clinic;<br>UNSW Global Pty Ltd;<br>Scientia Clinical Research Limited;<br>UNSW School of Psychology |
| **Insurer** | Newline Australia Insurance Pty Ltd |
| **Policy Number(s)** | AUS19888283A |
| **Period of Insurance** | From: 4.00 pm 01st November 2020 Local Standard Time<br>To: 4.00 pm 01st November 2021 Local Standard Time |
| **Limits of Liability** | **Coverage Section 3A: No Fault Compensation for Clinical Trials**<br>AUD 25,000,000 any **One Claim** and in the aggregate during the **Period of Insurance**<br>**Coverage Section 3B: Clinical Trials: Legal Liability**<br>AUD 25,000,000 any **One Claim** and in the aggregate during the **Period of Insurance**<br>**Coverage Section 4A: Medical Malpractice**<br>AUD 25,000,000 any **One Claim** and in the aggregate during the **Period of Insurance** |
| **Geographical Limit** | Australia |

**Further Information**
Should you have any queries, please contact us on the details set out at the top of the page.

**Important notes**
- Aon does not guarantee that the insurance outlined in this Certificate will continue to remain in force for the period referred to as the Policy may be cancelled or altered by either party to the contract, at any time, in accordance with the terms of the Policy and the Insurance Contracts Act 1984 (Clth).
- Aon accepts no responsibility or liability to advise any party who may be relying on this Certificate of such alteration to or cancellation of the Policy.
- Subject to full payment of premium
- This certificate does not:
  - represent an insurance contract or confer rights to the recipient; or
  - amend, extend or alter the Policy
  - contain the full policy terms and conditions

| | |
|---|---|
| **Retroactive Date** | 1 March 1997 other than 1 November 2017 in respect of $5,000,000 in excess of $20,000,000 |
| **Continuity Date** | 1 March 1997 other than 1 November 2017 in respect of $5,000,000 in excess of $20,000,000 |


**Further Information**

Should you have any queries, please contact us on the details set out at the top of the page.

**Important notes**

- Aon does not guarantee that the insurance outlined in this Certificate will continue to remain in force for the period referred to as the Policy may be cancelled or altered by either party to the contract, at any time, in accordance with the terms of the Policy and the Insurance Contracts Act 1984 (Clth).
- Aon accepts no responsibility or liability to advise any party who may be relying on this Certificate of such alteration to or cancellation of the Policy.
- Subject to full payment of premium
- This certificate does not:
  - represent an insurance contract or confer rights to the recipient; or
  - amend, extend or alter the Policy
  - contain the full policy terms and conditions
