# Supplementary material for: A mental health-informed, online health promotion programme targeting physical activity and healthy eating for adults aged 60+ years: study protocol for the MovingTogether randomised controlled trial
Source: Trials. 2022 Dec 27;23:1052. doi: 10.1186/s13063-022-06978-3 (PMC9793388; doi:10.1186/s13063-022-06978-3)
Supplement: Supplementary file 2 — Additional file 2:. Ethical Approval. [file 13063_2022_6978_MOESM2_ESM.pdf]

17-Sep-2021

Dear Associate Professor Simon Rosenbaum,

**Project Title**	Integrating StandingTall into a mental health informed online health promotion program for socially isolated adults aged 60 years old: the MovingTogether randomised controlled trial
**HC No**	HC210654
**Re**	HC210654 Notification of Ethics Approval
**Approval Period**	17-Sep-2021 - 16-Sep-2026

Thank you for submitting the above research project to the **HREC Executive** for ethical review. This project was considered by the **HREC Executive** at its meeting on **16-Sep-2021**.

I am pleased to advise you that the **HREC Executive** has granted ethical approval of this research project. The following condition(s) must be met before data collection commences:

Conditions of Approval:
N/A

Conditions of Approval - All Projects:

- The Chief Investigator will immediately report anything that might warrant review of ethical approval of the project.
- The Chief Investigator will seek approval from the **HREC Executive** for any modifications to the protocol or other project documents.
- The Chief Investigator will notify the **HREC Executive** immediately of any protocol deviation or adverse events or safety events related to the project.
- The Chief Investigator will report to the **HREC Executive** annually in the specified format and notify the **HREC Executive** when the project is completed at all sites.
- The Chief Investigator will notify the **HREC Executive** if the project is discontinued before the expected completion date, with reasons provided.
- The Chief Investigator will notify the **HREC Executive** of his or her inability to continue as Coordinating Chief Investigator including the name of and contact information for a

replacement.

The **HREC Executive** Terms of Reference, Standard Operating Procedures, membership and standard forms are available from https://research.unsw.edu.au/research-ethics-and-compliance-support-recs.

For questions or concerns, please contact the Ethics Administrator using the contact details below.
Telephone: Human Ethics team telephone contacts
Email: humanethics@unsw.edu.au
Website: https://research.unsw.edu.au/human-research-ethics-home

Kind Regards,

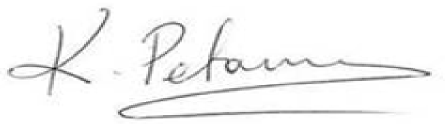

Associate Professor Kathy Petoumenos
Human Research Ethics Presiding Member

This HREC is constituted and operates in accordance with the National Health and Medical Research Council's (NHMRC) *National Statement on Ethical Conduct in Human Research (2007).* The processes used by this HREC to review multi-centre research proposals have been certified by the National Health and Medical Research Council.
