# Supplementary material for: A mental health-informed, online health promotion programme targeting physical activity and healthy eating for adults aged 60+ years: study protocol for the MovingTogether randomised controlled trial
Source: Trials. 2022 Dec 27;23:1052. doi: 10.1186/s13063-022-06978-3 (PMC9793388; doi:10.1186/s13063-022-06978-3)
Supplement: Supplementary file 3 — Additional file 3:. Participant Information Sheet. [file 13063_2022_6978_MOESM3_ESM.docx]

1. **What is the research study about?**

You are invited to take part in this research study which aims to evaluate an online physical activity program for adults aged over 60 years during the COVID-19 pandemic. You have been invited because you demonstrated an interest in participating in this study.

1. **Who is conducting this research?**

| The study is being carried out by the following researchers: | | |
| --- | --- | --- |
| **Role** | **Name** | **Organisation** |
| **Chief Investigator** | A/Prof Simon Rosenbaum | University of New South Wales |
| **Co-Investigator/s** | Ms Chiara Mastrogiovanni  Dr Grace McKeon  Prof Kim Delbaere  Prof Anne Tiedemann  Dr Scott Teasdale  PhD Candidate Annaliese McGavin  Prof Joel Pearson | University of New South Wales  University of New South Wales  University of New South Wales  University of Sydney  University of New South Wales  University of New South Wales  University of New South Wales |

1. **Inclusion/Exclusion Criteria**

Before you decide to participate in this research study, we need to ensure that it is okay for you to take part. The research study is looking recruit people who meet the following criteria:

1. 60+ years
2. Proficient in written and spoken English
3. Living in the community, in Australia
4. Have internet, a Facebook account (or able to set one up for the purpose of this study) and either computer, laptop or iPad/tablet access
5. Currently engaging in less than 150 mins of moderate-vigorous physical activity per week
6. Able to mobilise inside the house without the use of a walking aid
7. Have no medical conditions that exclude you from exercising safely
8. Not currently participating in a falls prevention program
9. Free of altered cognitive impairment or progressive neurological condition

Participants receiving a score above threshold in psychological distress (>30 in the Kessler 10) will be asked about their current mental health treatment and excluded if their medications have changed in the past 4 weeks.

Those scoring above threshold in suicidal ideation will be called by a psychologist for a suicide risk assessment.

1. **Do I have to take part in this research study?**

Participation in this research study is voluntary. If you do not want to take part, you do not have to. If you decide to take part and later change your mind, you are free to withdraw from the study at any stage. Your decision will not affect your relationship with The University of New South Wales.

If you decide you want to take part in the research study, you will be asked to:

- Read the information carefully (ask questions if necessary);
- Consent online via a secure website called REDCap (or over the phone with a research investigator if you prefer).

1. **What does participation in this research require, and are there any risks involved?**

This is a randomised controlled trial study which will be conducted over 10 weeks, with a pre, post and follow-up assessment where you will be asked a series of questionnaires. If you decide to participate in the research study, you will be asked to:

1. **Screening:** Screening questionnaires will ask about your physical activity, how you are feeling and your mental health and any health conditions you may have; this will determine if you are eligible to take part. Completing the screening measures will take approximately less than 10-25 minutes. The screening questionnaires will be administered to you via an email link to a website called REDCap (or verbally over the phone if you prefer).

REDCap is a secure web application used by UNSW researchers to collect data. If the screening questionnaire shows that you meet the criteria for inclusion, then you will be contacted with further details to take part in the study. If the screening questionnaires show that are not eligible, you will be notified via email and provided with some education resources on exercise and mental health.

1. **Randomisation:** The aim of the research is to compare the outcomes of the physical activity program and no program participation. To ensure that each participant has an equal chance of being placed in any group to start with, the REDCap website allocates each study participant into a group randomly, like the flip of a coin. All participants will be randomised to either the intervention group or the wait list control group. Both groups will have access to the program, however the difference between groups is that the wait list group will have a 14-week wait between completing initial questionnaires and beginning their 10-week program.
2. **Pre-assessment:** If you are eligible you will be complete a series of questionnaires prior to the program using an email link to REDCap (or verbally over the phone if you prefer) and a series of ‘Brain Games’ through a computer software called Inquisit 6. These activities will ask questions and allow you to participate in interactive activities that assess your mental health, quality of life, functional capacity, physical activity levels, cognition, social support and feelings of loneliness. This should take less than 30-45 minutes.
3. **Join the Facebook group**: You will be invited via email to join a private Facebook group specifically set-up for this study using the secret feature in Facebook which offers the highest level of security. The group will have approximately 30 members in total. If you don’t already have an account simply search ‘Facebook’ and follow the prompts on the main page under ‘create a new account’. It is suggested that you create a new Facebook account for the purpose of this study to protect your privacy from other participants. It is free. If you have any questions please contact the researcher.
4. **10-week intervention:** MovingTogether will be used to potentially improve both mental and physical health outcomes. To be involved in the group, participants will **l**og onto the Facebook group weekly or more often and read/participate in the content if you choose. Focus topics will include goal setting, barriers to exercise, nutrition and sedentary behaviour etc. Education will be provided by the exercise physiologists on pre-specified topics but there will also be room for open discussion on the group so you can receive feedback. You will also have the option to join weekly group video calls where the exercise physiologist will provide education on the different weekly topics. You can choose to join in the conversation or just listen. There is no need to sign up or login to the web portal. To join the call you can just click on the link which will be posted on the Facebook group. You can turn off the video if you’d prefer and just have voice enabled.

Participants will also gain access to the NeuRA’s StandingTall falls prevention and balance training program. This access will last beyond the 10-week program for a total of 24 months. StandingTall is a home-based, online, balance exercise program that aims to prevent falls. The exercises are tailored to your ability and specific needs. Users are guided through exercises with safety advice and instructional videos.

1. **Week 11 (post-assessment):** After the intervention, complete the series of questionnaires using REDCap (or over the phone if you prefer) and a series of ‘Brain Games’ through Inquisit 6. These activities will ask questions and allow you to participate in interactive activities that assess your mental health, quality of life, functional capacity, physical activity levels, cognition, social support and feelings of loneliness. This should take less than 30-45 minutes.
2. **Option to remain in the Facebook group and continue using StandingTall:** The group will no longer be managed by the researchers but you may continue to use it to provide and receive support from the other members. If participants choose to remain in the Facebook group or continue using the StandingTall app, researchers will not be providing ongoing support after the conclusion of the study.
3. **1-month follow up assessment:** After the intervention, complete the series of questionnaires using REDCap (or over the phone if you prefer) and a series of ‘Brain Games’ through Inquisit 6. These activities will ask questions and allow you to participate in interactive activities that assess your mental health, quality of life, functional capacity, physical activity levels, cognition, social support and feelings of loneliness. This should take less than 30-45 minutes.
4. **Optional Interview:** At the conclusion of the program you will be invited to participate in a 30-minute interview. This is optional and if you do not wish to participate in an interview, this will not affect your eligibility to participate in the program. If you choose to participate, you will be asked about your experience participating in the program and what you feel were the strengths and weaknesses of the program.

**Additional Costs and Reimbursement:** There are no costs associated with participating in this research project, nor will you be paid.

**Psychological Distress:** You may feel that some of the questions we ask are stressful or upsetting. If you do not wish to answer a question, you may skip it and go to the next question, or you may stop immediately. If you become upset or distressed as a result of your participation in the research project, the research team will be able to arrange for counselling or other appropriate support. Alternatively, a number of free contactable support services are included below. Any counselling or support will be provided by qualified staff who are not members of the research team. This counselling will be provided free of charge.

Please note that this is programme is not a mental health treatment program/emergency helpline and that the Facebook page will not be monitored 24/7. If however the questionnaires or any content posted on the Facebook group cause feelings of distress please use the contacts listed here:

- Lifeline- 131114

Online [www.lifeline.org.au](https://l.facebook.com/l.php?u=http%3A%2F%2Fwww.lifeline.org.au%2F&h=ATMtbdtxPbhjnMPpOQ3M8tpAzSGkuUaTZwEz203_ckGrkLvHtApb4rYXtPHhwCvD4UuGxlfHm3wY8EoNTiIDZlpmJ2aks4hUkQYERRiZgEcgks3xRq52hCyb) crisis chat (7pm to 4am)

- Beyondblue- 1300 22 4636

Online: [www.beyondblue.org.au](https://l.facebook.com/l.php?u=http%3A%2F%2Fwww.beyondblue.org.au%2F&h=ATMxhgL0-29wxXOtq7wlTkNeFm7tG_JAV7hM3lgpMHp5Iv2d07S7_WUzlzAFiY9LnUMoWmpuNrRpeJGbn_7_71x2_JXfYNFL7UEaWTOd-piBxnlVFYtPArQ_) (webchat 3pm to 12am)

1. **What are the possible benefits of taking part?**

We cannot guarantee or promise that you will receive any benefits from this research; however, possible benefits may include improved mental and physical health outcomes such as improvements in psychological distress and quality of life.

1. **What are the alternatives to taking part in the research?**

There is a small risk of injury with exercise however the program is designed by accredited exercise physiologists and you will be screened to exercise prior to commencement.

You can let the research team know if you have any concerns throughout the study and they will provide you with assistance. However, participation in this study is optional.

1. **What will happen to information about me?**

Submission of the online questionnaire (or verbal consent) is an indication of your consent. By clicking the ‘I agree to participate’ button (or verbally consenting via a phone call) you are providing your permission for the research team to collect and use information about you for the research study.

The research team will store the data collected from you for this research project for:

- A minimum of 15 years after the publication of the research results;

The information about you will be stored in a:

- Re-identifiable format where any identifiers such as your name, address, date of birth will be replaced with a unique code.

You will be asked to provide your consent for the research team to share or use the information collected from you in future research that:

- Will be used in any future research.

Your information will only be shared in a format that will not identify you.

- Information collected from you in an electronic format stored on a UNSW password protected OneDrive only accessible to the approved research investigators.

If you consent by clicking "I agree to participate" (or verbally consenting via a phone call) but do not complete the initial or subsequent questionnaires, the following process will occur:

1. Initial questionnaire: a single reminder will be sent via email before participants are considered lost to follow-up at the timing of the reminder
2. Subsequent questionnaires: an initial reminder via email will be sent followed by one follow up before participants are considered lost to follow-up at the timing of these reminders.

The information you provide is personal information for the purposes of the Privacy and Personal Information Protection Act 1998 (NSW). You have the right of access to personal information held about you by the University, the right to request correction and amendment of it, and the right to make a complaint about a breach of the Information Protection Principles as contained in the PPIP Act. Further information on how the University protects personal information is available in the [UNSW Privacy Management Plan](https://www.legal.unsw.edu.au/compliance/privacyhome.html).

1. **How and when will I find out what the results of the research study are?**

The research team intend to publish and/ report the results of the research. All Information will be published in a way that will not identify you. If you would like to receive a copy of the results you can let the research team know by inserting your email or mailing address in the consent form. We will only use these details to send you the results of the research.

1. **What if I want to withdraw from the research study?**

If you do consent to participate, you may withdraw at any time. You can do so by completing the ‘Withdrawal of Consent Form’ which is provided at the end of this document. Alternatively, you can ring the research team and tell them you no longer want to participate. Your decision not to participate or to withdraw from the study will not affect your relationship with UNSW Sydney or NeuRA. If you decide to leave the research study, the researchers will not collect additional information from you. You can request that any identifiable information about you be withdrawn from the research project.

1. **What if I have a complaint or any concerns about the research study and will I receive compensation if suffer any injuries or have complications?**

If you suffer any injuries or complications as a result of this research project, you should contact the study team as soon as possible and you will be assisted with arranging appropriate medical treatment. If you are eligible for Medicare, you can receive any medical treatment required to treat the injury or complication, free of charge, as a public patient in any Australian public hospital.

**Complaints Contact**

If you have a complaint regarding any aspect of the study or the way it is being conducted, please contact the UNSW Human Ethics Coordinator:

| **Position** | UNSW Human Research Ethics Coordinator |
| --- | --- |
| **Telephone** | + 61 2 9385 6222 |
| **Email** | [humanethics@unsw.edu.au](mailto:humanethics@unsw.edu.au) |
| **HC Reference Number** | HC210654 |

1. **What should I do if I have further questions about my involvement in the research study?**

The person you may need to contact will depend on the nature of your query. If you require further information regarding this study or if you have any problems which may be related to your involvement in the study, you can contact the following member/s of the research team:

**Research Team Contact**

| **Name** | Chiara Mastrogiovanni |
| --- | --- |
| **Position** | Masters of Research Candidate |
| **Telephone** | 9385 7536 |
| **Email** | c.mastrogiovanni@unsw.edu.au |

**Chief Investigator**

| **Name** | A/Prof Simon Rosenbaum |
| --- | --- |
| **Position** | Senior Research Fellow |
| **Telephone** | 9385 7536 |
| **Email** | s.rosenbaum@unsw.edu.au |

**Support Services Contact Details**

If at any stage during the study, you become distressed or require additional support from someone not involved in the research please call:

| **Name/Organisation** | Beyond Blue |
| --- | --- |
| **Position** | 1300 22 4636 |
| **Telephone** | [www.beyondblue.org.au](https://l.facebook.com/l.php?u=http%3A%2F%2Fwww.beyondblue.org.au%2F&h=ATMxhgL0-29wxXOtq7wlTkNeFm7tG_JAV7hM3lgpMHp5Iv2d07S7_WUzlzAFiY9LnUMoWmpuNrRpeJGbn_7_71x2_JXfYNFL7UEaWTOd-piBxnlVFYtPArQ_) (webchat 3pm to 12am) |

**Consent Form – Participant providing own consent**

**Declaration by the participant**

- I understand I am being asked to provide consent to participate in this research study;
- I have read the Participant Information Sheet, or someone has read it to me in a language that I understand;
- I understand the purposes, study tasks and risks of the research described in the study;
- I provide my consent for the information collected about me to be used for the purpose of this research study only.
- I have had an opportunity to ask questions and I am satisfied with the answers I have received;
- I freely agree to participate in this research study as described and understand that I am free to withdraw at any time during the study and withdrawal will not affect my relationship with any of the named organisations and/or research team members;
- I would like to receive a copy of the study results via email or post, I have provided my details below and ask that they be used for this purpose only;
- I understand that the results of the research will be made available once published in a peer-reviewed journal
- I would like to receive a copy of the study results via email or post, I have provided my details below and ask that they be used for this purpose only.

**Name: _____________________________________**

**Address: ___________________________________**

**Email Address: ______________________________**

Optional Consent for reuse of data and future research:

- I provide my consent for the information collected about me to made available to other researchers as described at section 8 of this document.

**Participant Signature**

| Name of Participant (please print) |  |
| --- | --- |
| Signature of Research Participant |  |
| Date |  |

**Declaration by Researcher***

- I have given a verbal explanation of the research study, its study activities and risks and I believe that the participant has understood that explanation.

**Researcher Signature***

| Name of Researcher (please print) |  |
| --- | --- |
| Signature of Researcher |  |
| Date |  |

^+^An appropriately qualified member of the research team must provide the explanation of, and information concerning the research study. All parties signing the consent section must date their own signature.

**Form for Withdrawal of Participation**

I wish to **WITHDRAW** my consent to participate in this research study described above and understand that such withdrawal **WILL NOT** affect my relationship with The University of New South Wales or NeuRA.

- I am withdrawing my consent and I would like any identifiable information collected about me which I have provided for the purpose of this research study withdrawn.
- I am withdrawing my consent to participate in further components of this research and provide my permission for the research team to retain and/or use information collected about me which I have provided for the purpose of this research.
- I am withdrawing my consent and I understand that any information already published and/or not linked to my identity cannot be withdrawn from the research.

**Participant Signature**

| Name of Participant  (please print) |  |
| --- | --- |
| Signature of Research Participant |  |
| Date |  |

**The section for Withdrawal of Participation should be forwarded to:**

| **Name** | Chiara Mastrogiovanni |
| --- | --- |
| **Position** | Masters of Research Candidate |
| **Telephone** | 9385 7536 |
| **Email** | c.mastrogiovanni@unsw.edu.au |
| **Post address** | Level 1, AGSM Building (G27), Gate 11, Botany St, UNSW Sydney Campus, Botany St, Kensington NSW 2052 |
